# Admission Plasma Lipopolysaccharide‐binding protein, Procalcitonin, and Lactate for Early Identification of Nosocomial Infection in Cirrhotic Patients With Upper Gastrointestinal Bleeding: A Retrospective Analysis

**DOI:** 10.1002/kjm2.70180

**Published:** 2026-01-23

**Authors:** Li Chen, Shan‐Shan Dun, Fang Xiao

**Affiliations:** ^1^ Department of Emergency Medicine Hubei NO. 3 People's Hospital of Jianghan University Wuhan China; ^2^ Department of Gastroenterology Hubei NO. 3 People's Hospital of Jianghan University Wuhan China

**Keywords:** cirrhosis, lipopolysaccharide—binding protein, nosocomial infection, procalcitonin, upper gastrointestinal bleeding

## Abstract

This study aimed to assess whether admission plasma lipopolysaccharide‐binding protein (LBP), procalcitonin (PCT), and lactate could improve detection of nosocomial infection in cirrhotic patients presenting with upper gastrointestinal bleeding (UGIB). A retrospective analysis was conducted in 196 consecutive adults with cirrhotic UGIB admitted between January 2021 and January 2025, in whom index biomarkers were defined as the first blood samples obtained within 24 h of hospital arrival and before any diagnosis of nosocomial infection. Nosocomial infection within 28 days of admission occurred in 58 of 196 patients. Compared with noninfected patients, those with nosocomial infection had higher WBC, CRP, PCT, LBP, lactate, international normalized ratio (INR), and total bilirubin (TB), lower albumin and sodium, a higher neutrophil‐to‐lymphocyte ratio (NLR), and a lower lymphocyte‐to‐monocyte ratio (LMR). Individual discrimination was excellent for LBP (area under the curve [AUC] 0.967), CRP (0.918), WBC (0.914), lactate (0.910), and PCT (0.901). In multivariable analysis, LBP, CRP, and albumin remained independently associated with nosocomial infection. A WBC + CRP model achieved an AUC of 0.975, whereas LBP + CRP + albumin and LBP + PCT + lactate panels yielded AUCs of 0.997 and 0.999, respectively; both LBP‐based panels significantly outperformed WBC + CRP. An admission LBP + CRP + albumin model provides a pragmatic, high‐performing tool for early risk stratification, while an LBP + PCT + lactate panel offers near‐perfect discrimination as an expanded option; these LBP‐based tri‐marker models may help refine early risk stratification and targeted management in cirrhotic patients with UGIB.

## 1 Introduction

Liver cirrhosis is the end‐stage consequence of chronic liver injury, marked by fibrosis, regenerative nodules, and distorted intrahepatic blood flow that culminate in hepatic dysfunction and portal hypertension [1]. It is a major global health burden and the 11th leading cause of death worldwide, accounting for 2.4% of deaths in 2019; in 2017 an estimated 112 million people had compensated and 10.6 million had decompensated cirrhosis, largely attributable to alcohol use disorder, HCV, and NAFLD/MASLD [1, 2, 3]. Upper gastrointestinal bleeding (UGIB) is a common reason for hospitalization and death among patients with cirrhosis, with variceal hemorrhage contributing to roughly 15% of cirrhosis‐related deaths annually [4].

Superimposed nosocomial infection further worsens hepatic inflammation and circulatory dysfunction, increasing the risks of rebleeding, hepatorenal syndrome, hepatic encephalopathy, and death, and prolonging length of stay and cost [5, 6]. These observations underscore the need for an objective, reproducible admission‐time biomarker‐based risk stratification tool to guide antimicrobial decisions, monitoring intensity, and reassessment frequency. In cirrhosis, infection pathophysiology is distinctive: portal hypertension and a compromised gut barrier promote bacterial translocation and increased endotoxin exposure, whereas systemic inflammation coexists with immunoparesis [7]. Under this biology, clinical signs and routine laboratory tests alone may offer limited discrimination, while pathway‐proximal biomarkers can add mechanistic and diagnostic value.

Lipopolysaccharide‐binding protein (LBP), a hepatocyte‐derived acute‐phase protein, rises in response to Gram‐negative lipopolysaccharide (LPS), forms LBP‐LPS complexes, and delivers LPS to the CD14/MD‐2/TLR4 axis, amplifying innate immune activation [8, 9]. In cirrhosis, circulating LBP reflects gut‐derived endotoxin burden and microbial translocation while also mirroring hepatic synthetic capacity, integrating both stimulus intensity and host response [10]. Procalcitonin (PCT) is upregulated across multiple tissues by bacterial toxins and inflammatory mediators and is generally more specific for bacterial than viral inflammation, providing information complementary to CRP, particularly in “bleeding plus possible infection” scenarios [11]. In liver disease, PCT retains diagnostic value for bacterial complications, though performance is population‐and threshold‐dependent [12, 13]. Lactate, a marker of tissue hypoperfusion and metabolic stress, is widely used as a severity indicator in sepsis [14]; in UGIB complicated by possible infection, elevated lactate signals systemic perfusion impairment or greater illness severity rather than infection per se [15].

Taken together, LBP (endotoxin/innate activation), PCT (bacterial specificity), and lactate (circulatory/metabolic severity) capture complementary axes spanning pathogen stimulus, host response, and systemic impact. It was therefore hypothesized that combined assessment of these markers at admission would provide more comprehensive early recognition of nosocomial infection risk than any single biomarker or routine test alone. Accordingly, the primary clinical objective of this study was to evaluate the diagnostic performance of a prespecified admission LBP + PCT + lactate panel, with secondary aims of comparing it with routine (WBC + CRP) and explanatory (LBP + CRP + albumin) models.

## 2 Methods and Materials

### 2.1 Ethics Statement

This retrospective study was approved by the institutional ethics committee, which granted a waiver of informed consent given the use of de‐identified data.

### 2.2 Participants

This single‐center retrospective cohort included consecutive adults (≥ 18 years) with cirrhosis admitted for UGIB between January 2021 and January 2025. Clinical data (demographics, comorbidities, medications, endoscopic therapy, and laboratory tests) were abstracted from the electronic medical records using a standardized form. Cirrhosis was confirmed by histology, characteristic imaging (ultrasound and/or transient elastography; mean liver stiffness ≥ 15 kPa), biochemical profile, or a combination thereof [16, 17, 18]. UGIB was confirmed clinically and by endoscopy in all cases. Peptic ulcer bleeding was treated with argon plasma coagulation or hypertonic saline‐epinephrine injection; variceal bleeding with endoscopic band ligation (overtube‐assisted) or cyanoacrylate injection. Prophylactic antibiotics were administered per institutional practice (e.g., oral ciprofloxacin 500 mg twice daily or intravenous ceftriaxone 2 g/day for ≥ 3 days) at the discretion of the treating physician. For the present analysis, prophylaxis data were additionally recorded, including whether each patient received prophylaxis, the main regimen used (fluoroquinolone vs. third‐generation cephalosporin vs. other), and the timing of initiation (within 24 h vs. > 24 h after admission). Exclusions were: insufficient evidence of cirrhosis; active infection present at admission (defined by clinical assessment supported by imaging and/or positive cultures obtained ≤ 7 days before admission); systemic antibiotics within 7 days before admission; malignancy; HIV infection, primary immunodeficiency, or prior organ transplantation. Fever and inflammatory markers (e.g., WBC, CRP) at admission were not used as standalone exclusion criteria and instead informed clinical adjudication of active infection when interpreted with other findings.

### 2.3 Definition of Nosocomial Infection

Nosocomial infection was ascertained from the medical record from ≥ 48 h after admission through hospital discharge or Day 28, whichever occurred first [19, 20]. Diagnosis followed standard clinical, microbiological, and imaging criteria and required at least one of the following: spontaneous bacterial peritonitis (SBP: ascitic polymorphonuclear leukocyte count ≥ 250 cells/mm^3^ irrespective of culture); urinary tract infection (UTI: leukocyturia and/or a positive urine culture meeting colony count thresholds); pneumonia (new pulmonary infiltrate on chest imaging plus compatible clinical features); bloodstream infection (BSI: positive blood cultures not judged as contamination with supportive clinical findings); or Clostridioides difficile infection (CDI: positive stool testing in a compatible clinical context). Patients with active infection at admission (before the 48‐h window) were excluded per protocol. In this cohort, 58 of 196 patients met the nosocomial infection definition and 138 did not.

### 2.4 Hematological/Biochemical Investigations at Admission

Admission laboratory data, defined as the first blood tests obtained within 24 h of hospital arrival and always before any diagnosis of nosocomial infection were extracted at presentation and included: white blood cells (WBC, 10^3^/μL), C‐reactive protein (CRP, μg/mL), sodium (Na, mmol/L), albumin (Alb, g/dL), total bilirubin (TB, mg/dL), hemoglobin (Hb, mg/dL), alkaline reserve (AR, mmol/L), platelet count (PLT, 10^3^/μL), platelet‐to‐lymphocyte ratio (PLR), neutrophil‐to‐monocyte ratio (NMR), lymphocyte‐to‐monocyte ratio (LMR), neutrophil‐to‐lymphocyte ratio (NLR), international normalized ratio (INR), erythrocyte sedimentation rate (ESR, mm/h), creatinine (Cr, mg/dL), aspartate aminotransferase (AST, U/L), and alanine aminotransferase (ALT, U/L). All assays were performed by the hospital laboratory under standard operating procedures.

### 2.5 Targeted Plasma Biomarkers (LBP, PCT, and Lactate)

At admission, venous blood was collected into EDTA tubes, processed to plasma after centrifugation at 4°C, and stored at −80°C until analysis. Plasma LBP was measured using a commercial single‐wash, ~90 min sandwich ELISA (Human LBP ELISA, SimpleStep, Abcam, ab279407) with optical‐density readout at 450 nm. Plasma PCT was quantified using a SimpleStep sandwich ELISA (Human Procalcitonin ELISA Kit, Abcam, ab221828). Lactate was determined by a colorimetric enzymatic assay (lactate dehydrogenase‐based) (l‐Lactate Assay Kit, Abcam, ab65331) per the manufacturer's protocol. Precision met prespecified criteria, with intra‐ and inter‐assay coefficients of variation < 10% and < 12%, respectively.

### 2.6 Statistical Methods

Statistical analyses were performed using Python (pandas, scikit‐learn, statsmodels) and R (pROC, rms). Continuous variables were compared primarily using the Mann–Whitney *U* test; Welch's *t*‐test was performed as a sensitivity analysis when approximate normality and unequal variances were plausible. For categorical variables, Fisher's exact test was used for 2 × 2 tables and Pearson's Chi‐square test for r × c tables. For diagnostic discrimination, receiver operating characteristic (ROC) curves were constructed and the area under the ROC curve (AUC) was estimated using DeLong's method. The optimal cut‐off was chosen by Youden's index. Sensitivity, specificity, positive predictive value (PPV), negative predictive value (NPV), and accuracy were calculated at the selected threshold. For combined diagnostics, a prespecified index biomarker model (LBP + PCT + lactate), a routine inflammatory benchmark model (WBC + CRP), and a pragmatic explanatory model (LBP + CRP + albumin) were evaluated at admission, and their predicted probabilities were compared using ROC analysis and DeLong's test. Binary outcomes were modeled with univariable and multivariable logistic regression. Multicollinearity was screened using variance inflation factors (VIFs) and pairwise Pearson correlations (flagged if VIF ≥ 5 or tolerance < 0.20). Two‐sided *p* < 0.05 was considered statistically significant. Statistical analyses were performed using Python 3.12.

## 3 Results

### 3.1 Baseline Characteristics

Among 196 patients, 58 (29.6%) developed nosocomial infection, with a median time to diagnosis of 6 days (IQR 3–8 days); among these 58 patients, the most frequent infection types were SBP (36.2%), UTI (25.9%), pneumonia (20.7%), BSI (13.8%), and CDI (3.4%). In the overall cohort, 177/196 (90.3%) of patients received prophylactic antibiotics. Third‐generation cephalosporins were the most commonly used regimen, accounting for 108/177 (61.0%), followed by fluoroquinolones in 55/177 (31.1%) and other or combination regimens in 14/177 (7.9%). Most patients started prophylaxis within 24 h of admission (154/177, 87.0%), whereas 23/177 (13.0%) initiated antibiotics after 24 h. Among culture‐positive cases (*n* = 46), Gram‐negative bacteria accounted for 29 isolates, Gram‐positive organisms for 14, and fungi for 3. The most frequently isolated Gram–negative pathogens were *Escherichia coli* (*n* = 13) and *Klebsiella* spp. (*n* = 7), followed by *Enterobacter* spp. (*n* = 3), *Pseudomonas aeruginosa* (*n* = 3), and other Gram‐negative rods (*n* = 3). Gram‐positive organisms were predominantly *Enterococcus* spp. (*n* = 6), *Staphylococcus aureus* (*n* = 3), coagulase‐negative staphylococci (*n* = 3), and *Streptococcus* spp. (*n* = 2), while fungi were mainly *Candida* spp. (3 isolates).

When stratified by nosocomial infection status (Table 1), age did not differ between the noninfected (*n* = 138) and infection (*n* = 58) groups (*p =* 0.739). Sex distribution was similar (*p* = 0.126), and the etiologies of cirrhosis did not differ between groups (*p* = 0.593). Child‐Pugh class showed a borderline overall difference (*p* = 0.072), with a higher proportion of class C in the infection group (48.3% vs. 31.2%). Bleeding types (esophageal or gastric variceal bleeding, gastric ulcer, duodenal ulcer) were comparable across groups (*p* = 0.470–0.866). The overall use of prophylactic antibiotics was similar between patients with and without nosocomial infection (91.4% vs. 89.9%), and the timing of initiation was likewise comparable (within 24 h: 92.5% vs. 84.7%).

**TABLE 1:** Baseline characteristics of the cohort, stratified by nosocomial infection status.

Variable	Infection (*n* = 58)	Noninfection (*n* = 138)	*p*
Age (years)	52.00 ± 12.35; 49.50 [44.25, 62.25]	52.57 ± 13.53; 52.00 [40.00, 65.00]	0.739
Gender			0.126
Male	8 (13.8%)	34 (24.6%)	
Female	50 (86.2%)	104 (75.4%)	
Cirrhosis etiology			0.593
Alcohol	32 (55.2%)	85 (61.6%)	
NASH	4 (6.9%)	11 (8.0%)	
Viral	22 (37.9%)	42 (30.4%)	
Child‐Pugh class			0.072
A	13 (22.4%)	38 (27.5%)	
B	17 (29.3%)	57 (41.3%)	
C	28 (48.3%)	43 (31.2%)	
Bleeding type			
Esophageal variceal	45 (77.6%)	111 (80.4%)	0.699
Gastric variceal	18 (31.0%)	41 (29.7%)	0.866
Gastric ulcer	12 (20.7%)	37 (26.8%)	0.470
Duodenal ulcer	1 (1.7%)	6 (4.3%)	0.676
Prophylactic antibiotics			1.000
Any prophylactic antibiotics	53 (91.4%)	124 (89.9%)	
None	5 (8.6%)	14 (10.1%)	
Type of prophylactic antibiotics			0.259
Third‐generation cephalosporins	37 (69.8%)	71 (57.3%)	
Fluoroquinolones	12 (22.6%)	43 (34.7%)	
Others	4 (7.5%)	10 (8.1%)	
Prophylaxis initiation time			0.223
Within 24 h of admission	49 (92.5%)	105 (84.7%)	
After 24 h of admission	4 (7.5%)	19 (15.3%)	

### 3.2 Admission Vital Signs and UGIB Symptoms

Vital signs were broadly comparable between groups (Table 2). Heart rate was 98.6 ± 20.1 (median 97.0 [79.2–118.5]) in the infection group vs. 95.1 ± 18.5 (93.5 [79.0–110.8]) in the noninfection group (*p* = 0.240). Systolic blood pressure was 116.7 ± 17.7 vs. 113.4 ± 17.1 (*p* = 0.245); diastolic blood pressure 75.2 ± 10.1 vs. 76.0 ± 11.3 (*p* = 0.607); temperature 36.5°C vs. 36.5°C (*p* = 0.562); respiratory rate 16.7 ± 1.9 vs. 17.1 ± 2.0 (*p* = 0.179). Presenting UGIB symptoms were similar: syncope 15.5% vs. 15.2% (*p* = 0.958), melena 51.7% vs. 55.1% (*p* = 0.668), hematemesis 56.9% vs. 62.3% (*p* = 0.478), coffee‐ground emesis 29.3% vs. 22.5% (*p* = 0.309).

**TABLE 2:** Admission vital signs and UGIB symptoms by nosocomial infection status.

Variable	Infection (*n* = 58)	Noninfection (*n* = 138)	*p*
Vital signs			
Heart rate	98.6 ± 20.1; 97.0 (79.2–118.5)	95.1 ± 18.5; 93.5 (79.0–110.8)	0.240
Systolic blood pressure	116.7 ± 17.7; 119.5 (100.5–131.0)	113.4 ± 17.1; 112.5 (99.0–128.0)	0.245
Diastolic blood pressure	75.2 ± 10.1; 73.0 (67.2–85.0)	76.0 ± 11.3; 76.0 (66.0–86.0)	0.607
Temperature (°C)	36.5 ± 0.3; 36.5 (36.3–36.8)	36.5 ± 0.3; 36.5 (36.2–36.8)	0.562
Respiratory rate	16.7 ± 1.9; 16.5 (15.0–18.0)	17.1 ± 2.0; 17.0 (15.0–19.0)	0.179
Symptoms of UGIB			
Syncope	9 (15.5%)	21 (15.2%)	0.958
Melena	30 (51.7%)	76 (55.1%)	0.668
Hematemesis	33 (56.9%)	86 (62.3%)	0.478
Coffee‐ground emesis	17 (29.3%)	31 (22.5%)	0.309

*Note:* Values are mean ± SD; median [IQR].

### 3.3 Laboratory Investigations at Admission

Compared with the non‐infection group, patients with nosocomial infection had higher WBC, CRP, PCT, LBP, and lactate (all *p* < 0.001), as well as higher total bilirubin and INR (both *p* < 0.001). Albumin and sodium were lower in the infection group (both *p* < 0.001). Blood—cell ratios were more unfavorable with higher NLR and lower LMR (both *p* < 0.001), while PLR was modestly higher (*p* = 0.003). Hemoglobin, alkaline reserve, platelet count, NMR, ESR, AST, creatinine, and ALT showed no statistically significant differences (all *p* > 0.05). Detailed summary statistics are provided in Table 3.

**TABLE 3:** Admission laboratory investigations comparing infection vs. noninfection groups.

Variable (unit)	Infection (*n* = 58)	Noninfection (*n* = 138)	*p*
WBC (10^3^/μL)	7.56 ± 1.32; 7.56 [6.45–8.47]	5.39 ± 0.87; 5.33 [4.68–6.10]	< 0.001
LBP (ng/mL)	91.75 ± 31.51; 89.63 [67.99–121.83]	35.43 ± 8.70; 34.70 [28.05–42.89]	< 0.001
CRP (μg/mL)	32.41 ± 8.80; 29.44 [25.21–40.40]	16.95 ± 7.04; 16.60 [10.73–23.00]	< 0.001
Na (mmol/L)	128.17 ± 7.86; 126.38 [122.97–133.98]	133.35 ± 7.94; 134.38 [129.84–137.89]	< 0.001
LMR	1.63 ± 0.64; 1.65 [0.99–2.17]	3.07 ± 1.25; 3.20 [1.97–4.06]	< 0.001
NLR	4.55 ± 2.36; 4.29 [2.23–6.90]	2.04 ± 0.85; 2.09 [1.36–2.70]	< 0.001
PCT (pg/mL)	630.41 ± 217.55; 635.80 [443.80–828.90]	303.08 ± 111.89; 310.35 [211.62–397.20]	< 0.001
Lac (mmol/L)	4.04 ± 0.84; 3.93 [3.30–4.89]	2.73 ± 0.45; 2.71 [2.33–3.16]	< 0.001
INR	2.14 ± 0.75; 2.21 [1.79–2.73]	1.66 ± 0.56; 1.63 [1.27–2.11]	< 0.001
Alb (g/dL)	2.25 ± 0.41; 2.32 [1.90–2.60]	2.88 ± 0.58; 2.90 [2.53–3.20]	< 0.001
TB (mg/dL)	4.48 ± 0.91; 4.34 [3.83–4.96]	2.89 ± 1.22; 2.95 [2.09–3.59]	< 0.001
PLR (ratio)	110.71 ± 53.60; 113.85 [60.55–151.60]	86.12 ± 33.67; 85.90 [58.17–116.90]	0.003
Hb (mg/dL)	10.68 ± 1.54; 10.61 [9.56–11.76]	11.26 ± 1.84; 11.14 [10.00–12.35]	0.066
AR (mmol/L)	19.94 ± 4.96; 19.25 [16.48–24.14]	21.15 ± 4.52; 21.05 [17.87–24.15]	0.111
PLT (10^3^/μL)	128.09 ± 50.89; 127.56 [90.32–159.40]	141.42 ± 61.25; 141.24 [102.23–188.31]	0.119
NMR	8.21 ± 5.70; 6.03 [3.61–13.36]	6.51 ± 3.77; 5.67 [3.75–8.93]	0.181
ESR (mm/h)	29.71 ± 13.44; 31.45 [17.07–38.07]	32.40 ± 13.16; 32.12 [23.93–41.55]	0.242
AST (U/L)	73.05 ± 27.48; 77.93 [54.54–91.50]	76.95 ± 34.80; 78.68 [49.31–101.28]	0.406
Cr (mg/dL)	1.26 ± 0.51; 1.25 [0.91–1.63]	1.36 ± 0.64; 1.37 [0.88–1.74]	0.451
ALT (U/L)	46.51 ± 25.46; 46.82 [25.05–62.98]	48.30 ± 25.09; 45.75 [30.28–63.53]	0.576

*Note:* Values are mean ± SD; median [IQR].

Abbreviations: Alb, albumin; ALT, alanine aminotransferase; AR, alkaline reserve; AST, aspartate aminotransferase; Cr, creatinine; CRP, C‐reactive protein; ESR, erythrocyte sedimentation rate; Hb, hemoglobin; INR, international normalized ratio; Lac, lactate; LBP, lipopolysaccharide‐binding protein; LMR, lymphocyte‐to‐monocyte ratio; Na, sodium; NLR, neutrophil‐to‐lymphocyte ratio; NMR, neutrophil‐to‐monocyte ratio; PCT, procalcitonin; PLR, platelet‐to‐lymphocyte ratio; PLT, platelet count; TB, total bilirubin; WBC, white blood cells.

### 3.4 Discriminative Performance of Individual Biomarkers

**FIGURE 1:**
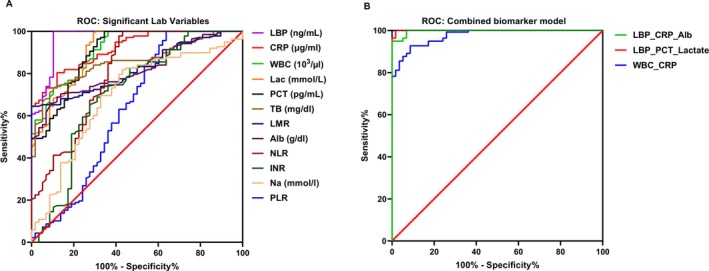
Receiver operating characteristic (ROC) curves for admission biomarkers and biomarker panels in predicting nosocomial infection in cirrhotic patients with upper gastrointestinal bleeding. (A) Individual biomarkers. ROC curves for selected admission biomarkers showing discriminative performance for nosocomial infection. (B) Comparison of ROC curves for three admission‐time biomarker models: The prespecified index panel LBP + PCT + lactate, the pragmatic explanatory model LBP + CRP + albumin derived from multivariable analysis, and a routine inflammatory benchmark model (WBC + CRP). Alb, albumin; AUC, area under the curve; CRP, C‐reactive protein; INR, international normalized ratio; LBP, lipopolysaccharide‐binding protein; LMR, lymphocyte‐to‐monocyte ratio; Na, sodium; NLR, neutrophil‐to‐lymphocyte ratio; PCT, procalcitonin; PLR, platelet‐to‐lymphocyte ratio; TB, total bilirubin; WBC, white blood cells.

ROC analyses prioritized markers that met two criteria: statistically and clinically meaningful between‐group differences and either AUC ≥ 0.70 or strong biologic plausibility. LBP showed an AUC of 0.967 (95% CI, 0.936–0.990), CRP 0.918 (0.879–0.950), WBC 0.914 (0.872–0.952), lactate 0.910 (0.861–0.951), and PCT 0.901 (0.848–0.944). TB performed well with an AUC of 0.856 (0.803–0.905). Lower LMR 0.821 (0.761–0.877) and lower albumin 0.816 (0.754–0.872) were associated with infection. NLR showed fair performance (AUC 0.803), while INR (AUC 0.707) and sodium (AUC 0.694) were borderline; PLR was weaker at AUC 0.637. Operating characteristics at Youden—optimal cut‐offs are summarized in Table 4 and Figure 1A.

**TABLE 4:** Diagnostic performance of individual biomarkers.

Marker	AUC (95% CI)	Sensitivity	Specificity	PPV	NPV	Accuracy
LBP (ng/mL)	0.967 (0.936–0.990)	90%	100%	100%	96%	97%
CRP (μg/mL)	0.918 (0.879–0.950)	88%	80%	65%	94%	83%
WBC (10^3^/μL)	0.914 (0.872–0.952)	64%	100%	100%	87%	89%
Lac (mmol/L)	0.910 (0.861–0.951)	71%	99%	98%	89%	91%
PCT (pg/mL)	0.901 (0.848–0.944)	66%	100%	100%	87%	90%
TB (mg/dL)	0.856 (0.803–0.905)	90%	73%	58%	94%	78%
LMR	0.821 (0.761–0.877)	100%	64%	54%	100%	75%
Alb (g/dL)	0.816 (0.754–0.872)	91%	66%	53%	95%	73%
NLR	0.803 (0.727–0.871)	57%	100%	100%	85%	87%
INR	0.707 (0.613–0.792)	64%	75%	51%	83%	71%
Na (mmol/L)	0.694 (0.610–0.777)	57%	82%	57%	82%	74%
PLR	0.637 (0.537–0.729)	36%	100%	100%	79%	81%

Abbreviations: Alb, albumin; CRP, C‐reactive protein; INR, international normalized ratio; Lac, lactate; LBP, lipopolysaccharide‐binding protein; LMR, lymphocyte‐to‐monocyte ratio; Na, sodium; NLR, neutrophil‐to‐lymphocyte ratio; PCT, procalcitonin; PLR, platelet‐to‐lymphocyte ratio; TB, total bilirubin; WBC, white blood cells.

### 3.5 Multivariable Analysis, Correlation, Multicollinearity, and Internal Validation

**FIGURE 2:**
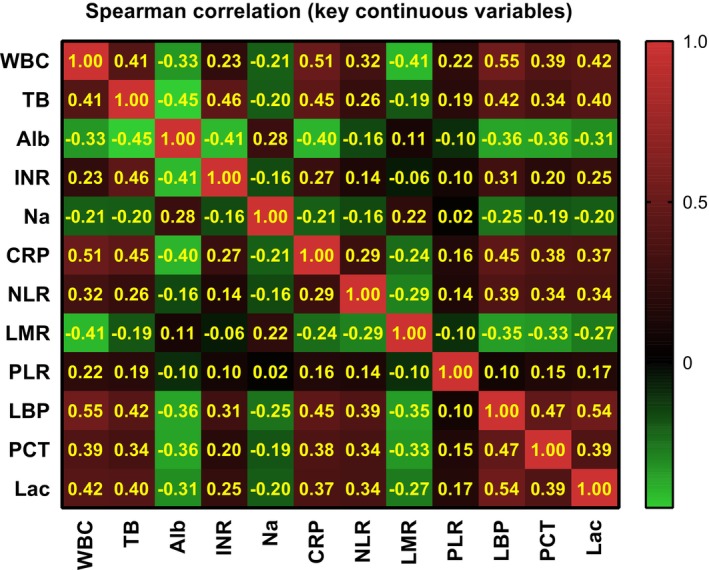
Pairwise correlations among candidate predictors at admission. Heatmap of pairwise Spearman correlation coefficients (*ρ*) among candidate predictors measured at admission. Correlations were modest overall, with all |*ρ*| < 0.60, indicating limited linear/monotonic association between variables. Alb, albumin; ALT, alanine aminotransferase; AST, aspartate aminotransferase; CRP, C‐reactive protein; ESR, erythrocyte sedimentation rate; Hb, hemoglobin; INR, international normalized ratio; LBP, lipopolysaccharide‐binding protein; LMR, lymphocyte‐to‐monocyte ratio; Na, sodium; NLR, neutrophil‐to‐lymphocyte ratio; NMR, neutrophil‐to‐monocyte ratio; PCT, procalcitonin; PLR, platelet‐to‐lymphocyte ratio; PLT, platelet count; TB, total bilirubin; WBC, white blood cells.

In univariate logistic regression (Table 5), higher WBC, LBP, CRP, NLR, PCT, lactate, INR, TB, and PLR, and lower albumin, sodium, LMR, and hemoglobin were associated with infection (all *p* < 0.05). Among clinical characteristics, Child‐Pugh class C vs. B was associated with increased risk (OR 2.18; 95% CI, 1.06–4.49). Vital signs, UGIB symptoms, and most routine chemistries (AST, ALT, ESR, creatinine, PLT, NMR) were not associated. The multivariable model retained three independent markers: LBP (adjusted OR 17.27; 95% CI, 11.72–25.12; *p* < 0.001), CRP (7.33; 4.77–10.83; *p* < 0.001), and albumin (0.42; 0.28–0.68; *p* < 0.001). Pairwise correlations among candidate predictors were modest (all absolute *r* < 0.60, Figure 2, Supplementary Table 1). In particular, LBP showed moderate positive correlations with WBC, CRP, lactate, and TB (*r* = 0.40–0.55; all *p* < 0.001), whereas albumin was inversely correlated with TB, CRP, LBP, and INR (*r* = −0.36 to −0.45; all *p* < 0.001). VIFs in the final model were low (range 1.20–1.47; tolerance 0.68–0.83). In the expanded candidate set, the highest VIF was 2.52, indicating no material multicollinearity.

**TABLE 5:** Logistic regression analyses: Univariable and multivariable predictors of nosocomial infection.

Variable	OR (95% CI)	*p*	OR (95% CI)	*p*
Gender (male vs. female)	0.489 (0.211–1.135)	0.126		
Age (years)	1.000 (0.974–1.019)	0.773		
Cirrhosis etiology				
Viral vs. alcohol	1.391 (0.721–2.683)	0.396		
NASH vs. alcohol	0.966 (0.287–3.254)	1.000		
Child‐Pugh				
A vs. B	1.147 (0.500–2.632)	0.832		
C vs. B	2.183 (1.062–4.490)	0.048		
Bleeding type				
Duodenal ulcer (yes vs. no)	0.386 (0.045–3.280)	0.676		
Esophageal variceal (yes vs. no)	1.188 (0.563–2.506)	0.699		
Gastric ulcer (yes vs. no)	0.712 (0.340–1.490)	0.470		
Gastric variceal (yes vs. no)	1.065 (0.547–2.071)	0.866		
Heart rate	1.010 (0.993–1.027)	0.239		
Systolic blood pressure	1.011 (0.993–1.030)	0.232		
Diastolic blood pressure	0.993 (0.967–1.020)	0.611		
Temperature (°C)	1.348 (0.521–3.483)	0.538		
Respiratory rate	0.898 (0.771–1.046)	0.168		
Symptoms of UGIB				
Coffee‐ground emesis (yes vs. no)	1.431 (0.716–2.860)	0.363		
Hematemesis (yes vs. no)	1.253 (0.672–2.337)	0.523		
Melena (yes vs. no)	1.144 (0.619–2.115)	0.754		
Syncope (yes vs. no)	1.023 (0.438–2.392)	1.000		
WBC (10^3^/μL)	7.987 (4.909–12.995)	< 0.001		
LBP (ng/mL)	1.243 (1.147–1.348)	< 0.001	17.269 (11.724–25.122)	< 0.001
CRP (μg/mL)	1.381 (1.275–1.496)	< 0.001	7.328 (4.768–10.833)	< 0.001
Na (mmol/L)	0.925 (0.887–0.965)	< 0.001		
LMR	0.283 (0.210–0.381)	< 0.001		
NLR	2.670 (2.089–3.413)	< 0.001		
PCT (pg/mL)	1.014 (1.010–1.017)	< 0.001		
Lac (mmol/L)	37.054 (14.334–95.785)	< 0.001		
INR	3.311 (1.742–6.295)	< 0.001		
Alb (g/dL)	0.103 (0.056–0.190)	< 0.001	0.424 (0.277–0.676)	< 0.001
TB (mg/dL)	3.622 (2.485–5.280)	< 0.001		
PLR	1.015 (1.006–1.023)	< 0.001		
Hb (mg/dL)	0.826 (0.695–0.981)	0.029		
AR (mmol/L)	0.945 (0.883–1.012)	0.108		
PLT (10^3^/μL)	0.996 (0.991–1.001)	0.118		
NMR	1.086 (1.015–1.163)	0.017		
ESR (mm/h)	0.985 (0.962–1.008)	0.191		
AST (U/L)	1.000 (0.988–1.005)	0.392		
Cr (mg/dL)	0.778 (0.488–1.240)	0.291		
ALT (U/L)	1.000 (0.985–1.010)	0.646		

Abbreviations: Alb, albumin; ALT, alanine aminotransferase; AR, alkaline reserve; AST, aspartate aminotransferase; Cr, creatinine CRP, C‐reactive protein; ESR, erythrocyte sedimentation rate; Hb, hemoglobin; INR, international normalized ratio; Lac, lactate; LBP, lipopolysaccharide‐binding protein; LMR, lymphocyte‐to‐monocyte ratio; Na, sodium; NLR, neutrophil‐to‐lymphocyte ratio; NMR, neutrophil‐to‐monocyte ratio; PCT, procalcitonin; PLR, platelet‐to‐lymphocyte ratio; PLT, platelet count; TB, total bilirubin; WBC, white blood cells.

### 3.6 Comparative Diagnostic Performance of Combined Biomarker Models

In exploratory analyses, the prespecified LBP + PCT + lactate panel was compared with a routine inflammatory model (WBC + CRP) and an explanatory LBP + CRP + albumin model for the discrimination of nosocomial infection (Figure 1B). All three combinations showed excellent discrimination in the overall cohort. The AUC was 0.975 (95% CI 0.943–0.992) for WBC + CRP, 0.997 (95% CI 0.975–1.000) for LBP + CRP + albumin, and 0.999 (95% CI 0.980–1.000) for LBP + PCT + lactate. Pairwise DeLong tests showed that both LBP + CRP + albumin and LBP + PCT + lactate significantly outperformed the WBC + CRP model (*p* = 0.012 and *p* = 0.005, respectively). In contrast, the difference between the two LBP‐based panels was small and not statistically significant (*p* = 0.177), indicating comparable diagnostic performance of LBP + CRP + albumin and LBP + PCT + lactate.

## 4 Discussion

Using admission‐time laboratory data, this study identified multiple biomarkers associated with nosocomial infection and showed that the prespecified three‐marker panel (LBP, PCT, lactate) delivered near‐perfect discrimination. These findings align with the current framework in cirrhosis whereby gut‐derived endotoxin, innate‐immune dysregulation, and circulatory/metabolic stress interact to heighten infection susceptibility [21]. Consistent with recent reviews and guidance, early and repeatable risk stratification is clinically valuable because infections precipitate acute decompensation and worsen outcomes [4, 22].

Among single markers, LBP yielded the highest AUC, consistent with its role as a hepatocyte‐derived acute‐phase protein that complexes with Gram‐negative LPS and drives CD14/MD‐2/TLR4 signaling to amplify innate responses; in cirrhosis, circulating LBP tracks both endotoxin burden and bacterial translocation, placing it closer to the causal pathway [10, 23, 24]. In interpreting these findings, it is important to recognize that LBP is not merely a marker of overt Gram‐negative infection but rather a surrogate of chronic endotoxin exposure and gut‐liver axis dysfunction in cirrhosis. LBP is a hepatocyte‐derived class I acute‐phase protein induced by bacterial lipopolysaccharide, and several studies in cirrhotic populations have proposed serum LBP as an indirect marker of bacterial translocation and low‐grade endotoxemia, with higher levels associated with more advanced portal hypertension, systemic inflammation, and an increased risk of infectious complications and mortality [10, 23, 25, 26]. In patients with portal hypertension and impaired intestinal barrier function, gut‐derived bacteria and endotoxin can translocate even when the clinically apparent infection is ultimately attributed to Gram‐positive or fungal pathogens. Against this background, elevated LBP in the present cohort is best interpreted as an integrated readout of endotoxin burden, bacterial translocation, and innate immune activation, rather than as a marker restricted to “pure” Gram‐negative infections. This conceptual framework helps explain why LBP retained excellent discriminatory performance despite the presence of Gram‐positive and fungal infections in our series. Accordingly, in cirrhotic patients with UGIB, LBP may be viewed as a global marker of endotoxin‐driven inflammation and infection susceptibility, and remains a reasonable infection marker even when the index pathogen isolated in culture is Gram‐positive.

PCT, generally more specific for bacterial than viral inflammation, demonstrates good diagnostic performance in cirrhosis and spontaneous bacterial peritonitis, typically complementing or exceeding CRP [27, 28]. Lactate, reflecting tissue hypoperfusion and inflammation‐driven metabolic reprogramming, contributes a severity dimension in the context of UGIB plus potential infection and therefore complements inflammatory markers.

Patterns among routine tests were coherent with biology. CRP, WBC, and TB showed strong‐to‐moderate discrimination, consistent with systemic inflammation and cholestasis/hepatocellular injury [29]. Low albumin and low LMR indicated higher risk, in line with reduced hepatic synthetic reserve and immune‐inflammatory imbalance and with associations to adverse outcomes in critical illness and sepsis [30]. NLR performed only fairly for diagnosis, though elevated NLR reliably associates with mortality and other adverse outcomes in cirrhosis (and cirrhosis with sepsis), suggesting greater utility for prognosis than for pure diagnostic separation [31]. INR and sodium offered borderline diagnostic value‐markers of underlying hepatic function and fluid homeostasis rather than infection, consistent with reports of limited sensitivity for acute infection recognition [21]. PLR was weaker, matching heterogeneous, population‐dependent findings in liver disease [32]. Taken together, WBC and CRP form a pragmatic, widely available benchmark combination that already provides excellent discrimination in this setting. Our LBP‐based panels should therefore be interpreted as incremental refinements over this WBC + CRP benchmark, offering statistically significant gains in diagnostic performance at the cost of additional, less routinely available assays.

In multivariable modeling, only LBP, CRP, and albumin remained independently associated, indicating that a three‐axis combination‐endotoxin/innate activation (LBP) + systemic inflammation (CRP) + hepatic synthetic reserve (albumin)‐captures most of the explainable signal and corresponds to core pathophysiology of infection susceptibility in cirrhosis [29]. At the same time, an operationally complementary approach using LBP (endotoxin/innate immunity), PCT (bacterial specificity), and lactate (circulatory/metabolic severity) can identify at admission those at high infection risk who may benefit from earlier antimicrobial coverage and closer monitoring, aligning with sepsis pathways that emphasize timely antibiotics and lactate‐guided assessment [27, 33]. Conventional laboratories (e.g., WBC, CRP, TB, albumin) then serve as host‐status background to be interpreted jointly with the more pathway–proximal or severity‐oriented markers [29].

From a clinical perspective, in a setting where prophylactic antibiotics are almost universally administered to cirrhotic patients with UGIB, the admission LBP + PCT + lactate panel should not be viewed as a gatekeeper for initiating prophylaxis but rather as a tool to refine early risk stratification. High biomarker profiles may justify intensified monitoring and a lower threshold for early escalation of antimicrobial therapy, whereas persistently low profiles under prophylaxis could, in future prospective studies, inform safe de‐escalation strategies. At present, these implications are hypothesis‐generating and do not supersede guideline‐based management.

This study has several limitations. First, it was a single‐center, retrospective analysis, so the estimates may reflect local case mix and care pathways and may not be generalizable to other settings. Second, the sample size and number of events (196 patients, 58 nosocomial infections) restrict the precision of effect estimates, particularly for multivariable models and threshold selection. Third, nosocomial infections were adjudicated by chart review of clinical, microbiological, and imaging documentation, which may have led to misclassification, especially for culture‐negative or partially treated episodes.

In addition, clinically apparent infection at admission was excluded, but nosocomial infection was defined as ≥ 48 h after admission; therefore, some early events may have represented incubating infections that were already influencing admission biomarkers, blurring the distinction between pure prediction of future infection and early detection of subclinical infection. Moreover, LBP, PCT, and lactate were implemented during the study period as part of the admission work‐up primarily in patients perceived to be at higher risk, so the cohort may over‐represent more severely ill patients, introducing spectrum and indication bias. Likewise, prophylactic antibiotics and subsequent escalation were prescribed at the discretion of treating physicians; although overall use and timing were broadly similar between groups, residual confounding by indication cannot be excluded. Finally, serial measurements of biomarkers and detailed data on antibiotic adherence and modification were not systematically collected, which precluded dynamic analyses of biomarker trajectories and treatment response.

## 5 Conclusion

In hospitalized cirrhotic patients with UGIB, an admission LBP + CRP + albumin model showed excellent discrimination and appears to be a pragmatic tool for early risk stratification to guide antibiotics and monitoring. The prespecified LBP + PCT + lactate index panel provided near‐perfect discrimination and may serve as an expanded diagnostic option where these assays are available. Both LBP‐based panels require multicenter prospective validation and calibration before routine implementation.

## Conflicts of Interest

The authors declare no conflicts of interest.

## Supporting information


**Supplementary Table 1:** Pairwise correlations among candidate predictors at admission.

## Data Availability

The data that support the findings of this study are available from the corresponding author upon reasonable request.
