# Supplementary material for: Admission Plasma Lipopolysaccharide‐binding protein, Procalcitonin, and Lactate for Early Identification of Nosocomial Infection in Cirrhotic Patients With Upper Gastrointestinal Bleeding: A Retrospective Analysis
Source: Kaohsiung J Med Sci. 2026 Jan 23:e70180. Online ahead of print. doi: 10.1002/kjm2.70180 (PMC13399873; doi:10.1002/kjm2.70180)
Supplement: Supplementary file 1 — Supplementary Table 1: Pairwise correlations among candidate predictors at admission. [file KJM2-9999-e70180-s001.docx]

**Supplementary Table 1 Pairwise correlations among candidate predictors at admission**

|  | | **Alb** | **CRP** | **LBP** | **INR** | **WBC** | **PCT** | **Lac** | **NLR** | **LMR** | **PLR** | **Na** | **TB** |
| --- | --- | --- | --- | --- | --- | --- | --- | --- | --- | --- | --- | --- | --- |
| **Alb** | *r* | 1 | -0.399 | -0.364 | -0.407 | -0.333 | -0.360 | -0.315 | -0.157 | 0.112 | -0.101 | 0.279 | -0.451 |
|  | ***P*** | 0 | **6.90E-09** | **1.50E-07** | **3.30E-09** | **1.90E-06** | **2.20E-07** | **7.10E-06** | **0.028** | 0.117 | 0.161 | **7.50E-05** | **3.10E-11** |
| **CRP** | *r* | -0.399 | 1 | 0.450 | 0.269 | 0.510 | 0.376 | 0.367 | 0.291 | -0.236 | 0.161 | -0.210 | 0.449 |
|  | ***P*** | **6.90E-09** | 0 | **3.70E-11** | **1.40E-04** | **2.30E-14** | **5.40E-08** | **1.20E-07** | **3.60E-05** | **0.001** | **0.024** | **0.003** | **4.00E-11** |
| **LBP** | *r* | -0.364 | 0.450 | 1 | 0.311 | 0.546 | 0.467 | 0.539 | 0.391 | -0.347 | 0.096 | -0.254 | 0.423 |
|  | ***P*** | **1.50E-07** | **3.70E-11** | 0 | **9.40E-06** | 0 | **5.00E-12** | **4.40E-16** | **1.40E-08** | **6.20E-07** | 0.180 | **3.20E-04** | **6.50E-10** |
| **INR** | *r* | -0.407 | 0.269 | 0.311 | 1 | 0.232 | 0.198 | 0.251 | 0.138 | -0.059 | 0.101 | -0.163 | 0.458 |
|  | ***P*** | **3.30E-09** | **1.40E-04** | **9.40E-06** | 0 | **1.00E-03** | **5.40E-03** | **3.90E-04** | 0.053 | 0.414 | 0.159 | **0.022** | **1.50E-11** |
| **WBC** | *r* | -0.333 | 0.510 | 0.546 | 0.232 | 1 | 0.386 | 0.423 | 0.321 | -0.405 | 0.217 | -0.209 | 0.406 |
|  | ***P*** | **1.90E-06** | **2.30E-14** | 0 | **0.001** | 0 | **2.30E-08** | **6.50E-10** | **4.40E-06** | **3.70E-09** | **0.002** | **0.003** | **3.50E-09** |
| **PCT** | *r* | -0.36 | 0.376 | 0.467 | 0.198 | 0.386 | 1 | 0.386 | 0.343 | -0.335 | 0.153 | -0.186 | 0.336 |
|  | ***P*** | **2.20E-07** | **5.40E-08** | **5.00E-12** | **0.005** | **2.30E-08** | 0 | **2.30E-08** | **8.50E-07** | **1.60E-06** | **0.032** | **0.009** | **1.40E-06** |
| **Lac** | *r* | -0.315 | 0.367 | 0.539 | 0.251 | 0.423 | 0.386 | 1 | 0.343 | -0.270 | 0.172 | -0.204 | 0.403 |
|  | ***P*** | **7.10E-06** | **1.20E-07** | **4.40E-16** | **3.90E-04** | **6.50E-10** | **2.30E-08** | 0 | **8.80E-07** | **1.30E-04** | **0.016** | **0.004** | **4.70E-09** |
| **NLR** | *r* | -0.157 | 0.291 | 0.391 | 0.138 | 0.321 | 0.343 | 0.343 | 1 | -0.288 | 0.137 | -0.162 | 0.256 |
|  | ***P*** | **0.028** | **3.60E-05** | **1.40E-08** | 0.053 | **4.40E-06** | **8.50E-07** | **8.80E-07** | 0 | **4.10E-05** | 0.056 | **0.023** | **2.89E-04** |
| **LMR** | *r* | 0.112 | -0.236 | -0.347 | -0.059 | -0.405 | -0.335 | -0.270 | -0.288 | 1 | -0.103 | 0.220 | -0.190 |
|  | ***P*** | 0.117 | **8.60E-04** | **6.20E-07** | 0.414 | **3.70E-09** | **1.60E-06** | **1.30E-04** | **4.10E-05** | 0 | 0.151 | **0.002** | **0.008** |
| **PLR** | *r* | -0.101 | 0.161 | 0.096 | 0.101 | 0.217 | 0.153 | 0.172 | 0.137 | -0.103 | 1 | 0.020 | 0.195 |
|  | ***P*** | 0.161 | **0.024** | 0.180 | 0.159 | **0.002** | **0.032** | **0.016** | 0.056 | 0.151 | 0 | 0.780 | **0.006** |
| **Na** | *r* | 0.279 | -0.210 | -0.254 | -0.163 | -0.209 | -0.186 | -0.204 | -0.162 | 0.220 | 0.020 | 1 | -0.203 |
|  | ***P*** | **7.50E-05** | **0.003** | **3.20E-04** | **0.022** | **0.003** | **0.009** | **0.004** | **0.023** | **0.002** | 0.780 | 0 | **0.004** |
| **TB** | *r* | -0.451 | 0.449 | 0.423 | 0.458 | 0.406 | 0.336 | 0.403 | 0.256 | -0.190 | 0.195 | -0.203 | 1 |
|  | ***P*** | **3.10E-11** | **4.00E-11** | **6.50E-10** | **1.50E-11** | **3.50E-09** | **1.40E-06** | **4.70E-09** | **2.90E-04** | **0.008** | **0.006** | **0.004** | 0 |

Note: Alb, albumin; CRP, C-reactive protein; INR, international normalized ratio; LBP, lipopolysaccharide-binding protein; LMR, lymphocyte-to-monocyte ratio; NLR, neutrophil-to-lymphocyte ratio; Na, sodium; PCT, procalcitonin; PLR, platelet-to-lymphocyte ratio; PLT, platelet count; TB, total bilirubin; WBC, white blood cells. Statistically significant correlations (P < 0.05) are shown in bold.
